# Non-Close-Packed Isotropic Responsive Magnetic Photonic Crystal Microspheres

**DOI:** 10.3390/nano16090556

**Published:** 2026-05-01

**Authors:** Lejian Zhao, Jie Zhu, Maocheng Sun, Wei Luo, Huiru Ma, Jianguo Guan

**Affiliations:** 1 State Key Laboratory of Advanced Technology for Materials Synthesis and Processing, International School of Materials Science and Engineering, Wuhan University of Technology, Wuhan 430070, China; 298552@whut.edu.cn (L.Z.); guanjg@whut.edu.cn (J.G.); 2 School of Materials Science and Engineering, Wuhan University of Technology, Wuhan 430070, China; 345097@whut.edu.cn (J.Z.); 364357@whut.edu.cn (M.S.); 3 School of Chemistry, Chemical Engineering and Life Science, Wuhan University of Technology, Wuhan 430070, China; 4 Wuhan Institute of Photochemistry and Technology, 7 North Bingang Road, Wuhan 430083, China

**Keywords:** magnetic photonic crystal microspheres, non-close-packed architecture, angle-independent coloration, stimuli-responsive hydrogels

## Abstract

Magnetic photonic crystal microspheres (MPCMs) have emerged as a versatile platform for intelligent sensing and display applications, owing to their integration of magnetic actuation with structural coloration. However, their practical implementation is limited by a fundamental structural constraint: most reported MPCMs adopt anisotropic architectures, resulting in angle-dependent optical responses that require continuous magnetic alignment to maintain uniform coloration. Herein, we propose a different structural paradigm based on non-close-packed, optically isotropic MPCMs. Driven by electrostatic repulsion in solutions, monodisperse Fe_3_O_4_@tannic acid (TA) core–shell nanoparticles spontaneously assemble into non-close-packed amorphous colloidal arrays, also known as photonic glasses, which are subsequently immobilized within stimuli-responsive polymer networks via emulsification-assisted thermal polymerization. By integrating poly(2-hydroxyethyl methacrylate-*co*-*N*-vinylpyrrolidone) (HEMA–NVP) or poly(*N*-isopropylacrylamide) (PNIPAM) as responsive matrices, the resulting MPCMs exhibit sensitive solvent- or thermo-dependent optical responses. Crucially, structural isotropy ensures angle-independent coloration, eliminating the need for continuous magnetic alignment during optical readout. As evidenced by the unchanged structural color and reflection peak under various magnetic field orientations, this design effectively decouples optical sensing from magnetic actuation. The intrinsic free volume of the non-close-packed architecture allows for isotropic lattice expansion and contraction, leading to broad spectral tunability. Collectively, this work establishes a promising design framework for magnetic photonic microsensors.

## 1. Introduction

Photonic crystals (PCs) are periodic dielectric architectures that regulate light propagation through photonic band gaps, producing structural colors that are intrinsically stable, spectrally selective, and resistant to photobleaching [1,2,3]. When coupled with stimuli-responsive polymer matrices, dynamic variations in lattice spacing or effective refractive index can be directly transduced into visible optical signals, establishing a robust platform for tunable photonic applications [4,5]. Magnetic core–shell nanoparticles and smart photonic architectures for structural coloration and multi-stimuli responsiveness have been extensively developed, including immobilized magnetochromatic structures for color printing [6,7,8], angle-independent structural color materials [9,10,11,12], and responsive sensing [12,13,14], among others [15]. Accordingly, the incorporation of superparamagnetic nanoparticles further extends this concept, leading to the emergence of magnetic photonic crystal microspheres (MPCMs) that integrate optical readout with magnetic manipulability [16,17]. Unlike conventional planar films, the spherical geometry of MPCMs inherently offers higher specific surface area, rapid mass-transport kinetics, and excellent dispersion stability [18,19]. These attributes, combined with their compatibility with scalable droplet microfluidics and emulsion fabrication methods, render them highly attractive for high-throughput applications [16,20]. By harnessing non-contact magnetic manipulation, targeted spatial enrichment, and direct visual detection, MPCMs have emerged as promising candidates in diverse fields, including multiplexed bioanalysis [21], anti-counterfeiting [22], and intelligent displays [23,24].

However, the practical deployment of current MPCM systems is severely constrained by intrinsic structural limitations in their design and assembly mechanisms. To integrate magnetic and optical functions without mutual interference, existing designs predominantly utilize anisotropic configurations, such as Janus-type compartmentalized microspheres [25,26] or one-dimensional chain-like magnetic assemblies [27]. As a result, the structural colors of MPCMs are inherently angle-dependent and require continuous and precise magnetic alignment for reliable optical readout. This stringent operational requirement not only increases system complexity and energy consumption, but also critically undermines signal stability, particularly in dynamic fluidic conditions or heterogeneous microenvironments where even slight orientational deviations can lead to significant optical fluctuations or complete signal loss [28,29]. Beyond these operational challenges, the majority of reported MPCMs are constructed from close-packed colloidal lattices characterized by rigid interparticle contacts. Such densely packed structural frameworks impose strong steric constraints on the responsive polymer matrix, which can effectively restrict lattice contraction or reduce the magnitude of volume change. Consequently, the volume phase transitions of the hydrogel are limited to lattice expansion that severely narrows the tunable range of the photonic stopband and diminishes the overall sensitivity to external chemical or physical stimuli [30,31]. While non-close-packed photonic crystals have been extensively investigated for their potential in stimuli-responsive applications [32] and amorphous photonic structures assembled from magnetic particles have been immobilized or encapsulated as thin films to achieve angle-independent structural coloration [11,33,34], the integration of these features with magnetic responsiveness in a microsphere format remains largely unexplored.

In this work, monodisperse Fe_3_O_4_@tannic acid (TA) core–shell nanoparticles are employed as building blocks. Their spontaneous assembly into non-close-packed colloidal arrays is governed by multiple factors, including the high negative ζ-potential (−60.2 mV, Figure S1) of the nanoparticles, high solvent polarity provided by NVP, optimized particle concentration, and low ionic strength. These optically isotropic arrays are subsequently immobilized in situ via mechanical emulsification-assisted thermal polymerization. By integrating poly(2-hydroxyethyl methacrylate-*co*-*N*-vinylpyrrolidone) (HEMA–NVP) and poly(*N*-isopropylacrylamide) (PNIPAM) as representative responsive matrices, we successfully constructed MPCMs exhibiting highly sensitive solvent- and thermo- responsive optical behaviors. Unlike close-packed systems, this flexible non-close-packed network accommodates both unrestricted expansion and contraction, affording a broad spectral tuning range. Crucially, the resulting MPCMs display angle-independent structural colors without the need for external magnetic alignment, thereby enabling passive and stable optical readout. Meanwhile, their inherent superparamagnetism allows for rapid, on-demand magnetic separation and targeted positioning, successfully decoupling optical sensing from magnetic actuation, and providing a framework for promising intelligent microsensors.

## 2. Materials and Methods

### 2.1. Materials

*N*-Vinyl-2-pyrrolidone (NVP, ≥99%), 2-hydroxyethyl methacrylate (HEMA, ≥96%), ethoxylated trimethylolpropane triacrylate (ETPTA, *M*_n_~428 g/mol), *N*-isopropylacrylamide (NIPAM, ≥98%), 2,2′-azobis(2,4-dimethylvaleronitrile) (ADVN, ≥98%), sodium dodecyl sulfate (SDS, ≥98%), acetonitrile (ACN, ≥99%), acetone (≥99.5%), tetrahydrofuran (THF, ≥99%), and pyridine (≥99.5%) were purchased from Aladdin Reagent Co., Ltd. (Shanghai, China). Methanol (≥99.5%), ethanol (≥99.7%), ethylene glycol (EG, ≥99.5%), and dimethyl sulfoxide (DMSO, ≥99.5%) were purchased from Sinopharm Chemical Reagent Co., Ltd. (Shanghai, China). Polydimethylsiloxane (PDMS, viscosity ~5000 mPa·s at 25 °C), propylene carbonate (PCb, ≥99.7%), and *N*,*N*-dimethylformamide (DMF, ≥99.5%) were purchased from Macklin Biochemical Co., Ltd. (Shanghai, China). All chemicals were used as received without further purification. Deionized water was produced in a Milli-Q system (Millipore, Burlington, MA, USA). Fe_3_O_4_ nanoparticles were synthesized according to our previous work [35]. The obtained Fe_3_O_4_ nanoparticles were then coated with tannic acid (TA) following a reported method to afford Fe_3_O_4_@TA core–shell nanoparticles [11]. The resulting Fe_3_O_4_@TA nanoparticles were dispersed in ethanol as a stable brown-black colloidal dispersion with a concentration of 20 mg/mL and stored at room temperature for subsequent use. Detailed experimental procedures are provided in the Supplementary Information (Section S1). The chemical structures of the polymers used in this work (P(HEMA-*co*-NVP) and PNIPAM) are shown in Figure S2.

### 2.2. Preparation of Solvent-Responsive MPCMs

First, 1 mL of the Fe_3_O_4_@TA ethanol dispersion (20 mg/mL) was centrifuged, the supernatant was discarded, and the precipitate (20 mg) was mixed with 82 μL of NVP, 35 μL of HEMA, 2 μL of ETPTA, and 1 mg of ADVN in a vial. The mixture was ultrasonicated until a homogeneous dispersion with a total volume of approximately 120 μL was obtained. Subsequently, the dispersion was transferred into a 10 mL glass beaker containing 2 mL of PDMS (viscosity of 5000 mPa·s), giving a volume ratio of dispersion to PDMS of 3:50. The mixture was stirred at 160 rpm for 5 min using a micro-stirrer (HUXI, Shanghai, China) fitted with a two-blade PTFE impeller (shaft diameter: 5 mm, impeller diameter: 15 mm, positioned close to the bottom of the beaker), during which the dispersion was broken into micrometer-sized droplets under the applied shear force. After stirring, the beaker was placed in an oven preheated to 80 °C and thermally cured for 5 min to obtain MPCMs. After curing, 4 mL of SDS aqueous solution (2 wt%) was added, and the mixture was ultrasonicated for 10 min to emulsify and remove the PDMS. The mixture was then centrifuged, and the supernatant (containing the emulsified PDMS) was discarded. The MPCMs were washed three times with deionized water and three times with ethanol. For each wash, the MPCMs were collected by centrifugation, and the supernatant was discarded. Finally, the obtained MPCMs were dispersed in 1 mL of ethanol and stored for further use.

### 2.3. Preparation of Thermo-Responsive MPCMs

First, 1 mL of the Fe_3_O_4_@TA ethanol dispersion (20 mg/mL) was centrifuged, the supernatant was discarded, and the precipitate (20 mg) was mixed with 75 μL of PCb, 42 mg of NIPAM, 2 μL of ETPTA, and 1 mg of ADVN in a vial. The mixture was ultrasonicated until a homogeneous dispersion with a total volume of approximately 120 μL was obtained. Subsequently, the dispersion was transferred into a 10 mL glass beaker containing 2 mL of PDMS (viscosity of 5000 mPa·s), giving a volume ratio of dispersion to PDMS of 3:50. The mixture was stirred at 160 rpm for 5 min using a micro-stirrer (HUXI, Shanghai, China) fitted with a two-blade PTFE impeller (shaft diameter: 5 mm, impeller diameter: 15 mm, positioned close to the bottom of the beaker), during which the dispersion was broken into micrometer-sized droplets under the applied shear force. After stirring, the beaker was placed in an oven preheated to 80 °C and thermally cured for 5 min to obtain MPCMs. After curing, 4 mL of SDS aqueous solution (2 wt%) was added, and the mixture was ultrasonicated for 10 min to emulsify and remove the PDMS. The mixture was then centrifuged, and the supernatant (containing the emulsified PDMS) was discarded. The MPCMs were washed three times with deionized water and three times with ethanol. For each wash, the MPCMs were collected by centrifugation, and the supernatant was discarded. Finally, the obtained MPCMs were dispersed in 1 mL of ethanol and stored for further use.

### 2.4. Characterizations

The morphology and structure of the samples were characterized using a field emission scanning electron microscope (FE-SEM, Hitachi S-4800, Tokyo, Japan). Fourier transform infrared (FTIR) spectra were recorded on a Nicolet 6700 spectrometer (Thermo Fisher Scientific, Waltham, MA, USA) in the range of 650–4000 cm^−1^ with a resolution of 4 cm^−1^. Thermogravimetric analysis (TGA) was performed on a NETZSCH STA 2500 Regulus thermal analyzer (NETZSCH, Yokohama, Japan) from room temperature to 1000 °C at a heating rate of 5 °C/min under an air atmosphere. The ζ-potential was measured in ethanol using a Zetasizer 3000HSA (Malvern Panalytical, Malvern, UK). Magnetic properties were measured using a vibrating sample magnetometer (VSM, Lake Shore 7404, Lake Shore Cryotronics, Westerville, OH, USA) at room temperature. Optical microscopy images were captured using an optical microscope (Zeiss Axio Observer 5 M, Zeiss, Göttingen, Germany). Reflection spectra were recorded with a fiber optic spectrometer (USB2000+, Ocean Optics, Shanghai, China) coupled to the optical microscope. For measurements requiring dry-state samples (e.g., FTIR, TGA, VSM), the MPCMs were vacuum-dried at 60 °C for 12 h prior to analysis.

## 3. Results and Discussion

**Figure 1:**
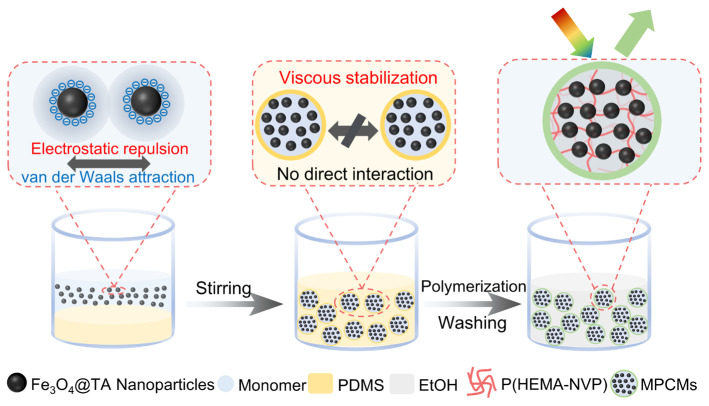
Schematic illustration of the preparation of MPCMs.

Figure 1 illustrates the preparation of magnetic photonic crystal microspheres (MPCMs) encapsulating non-close-packed Fe_3_O_4_ nanoparticle arrays. Initially, Fe_3_O_4_@TA core–shell nanoparticles are uniformly dispersed into a mixture comprising 2-hydroxyethyl methacrylate (HEMA), *N*-vinylpyrrolidone (NVP), and the thermal initiator 2,2′-Azobis(2,4-dimethylvaleronitrile) (ADVN). The electronegative carbonyl oxygen atoms within the NVP monomers exhibit a strong affinity for the protons of the phenolic hydroxyl groups on the TA shell. This specific intermolecular interaction induces an interfacial polarization phenomenon, effectively promoting the deprotonation of TA and endowing the Fe_3_O_4_@TA nanoparticles with a strong negative surface charge. Benefiting from this high negative ζ-potential and the polar solvent environment provided by NVP (which possesses a high dielectric constant), an electrical double layer is established around each particle. A control experiment without NVP (using only HEMA) yielded microspheres with no structural color when dispersed in ethanol (Figure S3), confirming that NVP is essential for establishing the high negative surface charge and the subsequent non-close-packed assembly. Consequently, this electrostatic interaction drives the spontaneous self-assembly of the nanoparticles into a thermodynamically stable non-close-packed colloidal structure.

Subsequently, the dispersion containing these dynamically stabilized arrays is introduced into a continuous fluidic phase consisting of high-viscosity PDMS (5000 mPa·s). Under mechanical stirring, hydrodynamic shear forces fragment the bulk aqueous dispersion into spherical droplets with an average diameter of 29.6 μm and a coefficient of variation (CV) of 28.7% (Figure S4), thereby generating a stable water-in-oil (W/O) emulsion. Notably, the high viscosity of the continuous PDMS phase serves a dual protective function. First, it restricts the Brownian mobility of the dispersed microdroplets, reducing their collision frequency. Second, it retards the drainage of the continuous oil film between two approaching droplets, effectively suppressing both droplet coalescence and aggregation. This ensures dispersion stability of the emulsion system prior to crosslinking. The curing time at 80 °C was optimized by testing 3, 5, and 7 min. Optical microscopy images of the microspheres dispersed in ethanol (Figure S5) showed that after 3 min, the microspheres exhibited irregular shapes and poorly defined edges, indicating incomplete polymerization. After 5 min, fully crosslinked microspheres with intact spherical morphology were obtained. Extending the curing time to 7 min did not cause further improvement. Therefore, a curing time of 5 min was selected. Upon elevating the temperature of the emulsion, the initiator undergoes thermal decomposition, triggering the crosslinking copolymerization of HEMA and NVP within the microdroplet reactors [36]. The consequent rapid in situ gelation forms a hydrogel network that permanently immobilizes the non-close-packed colloidal arrays.

**Figure 2:**
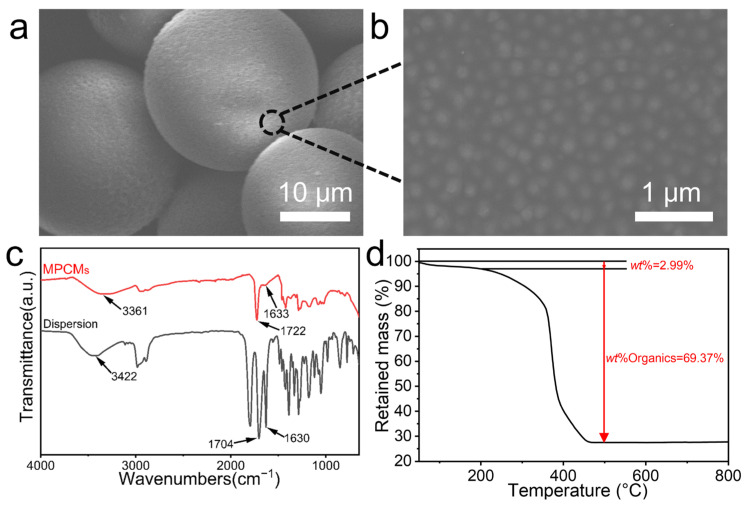
Morphological and compositional characterization of MPCMs. (**a**) SEM image of MPCMs. (**b**) Higher-magnification SEM image of the same MPCMs inset of (**a**). (**c**) FTIR spectra of dispersion and MPCMs. (**d**) TGA curves of MPCMs.

The Fe_3_O_4_@TA nanoparticles used as building blocks exhibit a spherical morphology with an average diameter of 148 nm (Figure S6). As revealed by the scanning electron microscopy (SEM) images in Figure 2a, the as-obtained MPCMs display a spherical morphology. Statistical analysis of optical microscopy images of MPCMs dispersed in ethanol (Figure S7) gave an average diameter of 19.8 μm with a CV of 27.8%. Further magnified observation (Figure 2b) confirms that these nanoparticles are assembled into a non-close-packed structure within the MPCMs, with a measured center-to-center distance between adjacent nanoparticles of 233.9 ± 27.5 nm (Figure S8), which is significantly larger than the particle diameter (148 nm), confirming the absence of direct interparticle contact. In the Fourier transform infrared (FTIR) spectrum of the dispersion (Figure 2c), a distinct characteristic absorption peak at 1630 cm^−1^ is assigned to the stretching vibrations of the acrylate C=C bonds in HEMA and the vinyl C=C bonds in NVP. After polymerization, this peak attenuates and shifts slightly to 1633 cm^−1^, becoming almost indiscernible. Simultaneously, the strong absorption band at 1704 cm^−1^, corresponding to the stretching vibrations of the ester C=O in HEMA and the amide C=O in NVP, shifts to 1722 cm^−1^ in the cured MPCMs without any loss in intensity. These combined spectral results verify the highly efficient crosslinking copolymerization of HEMA and NVP. Furthermore, the broad absorption peak at 3422 cm^−1^ primarily originates from the -OH stretching vibrations of both the HEMA monomers and the phenolic hydroxyl groups on the Fe_3_O_4_@TA nanoparticles. After curing, this peak shifts to a lower wavenumber of 3361 cm^−1^. This 61 cm^−1^ redshift indicates that the hydroxyl groups form stronger hydrogen bonds after crosslinking, consistent with the formation of hydrogen-bonding networks between the Fe_3_O_4_@TA nanoparticles and the polymer matrix.

The compositional ratio of the composite MPCMs was quantitatively assessed via thermogravimetric analysis (TGA) (Figure 2d). The initial minor mass loss of approximately 2.99% from 0 to 200 °C is primarily attributed to the evaporation of the adsorbed solvent. In the range of 200–500 °C, a drastic weight loss of 69.37% occurs, corresponding to the oxidative degradation and decomposition of the covalently crosslinked HEMA–NVP hydrogel network. Upon further heating beyond 500 °C, the mass curve exhibits a slight upward trend, which originates from the thermal oxidation of Fe_3_O_4_ into α-Fe_2_O_3_, leaving a residual mass of 27.64%. For reference, TGA of the pristine Fe_3_O_4_@TA nanoparticles (Figure S9) shows an inorganic fraction of 77.28% from the Fe_3_O_4_ core. Based on this value, the Fe_3_O_4_@TA nanoparticles are estimated to account for approximately 35.8% of the MPCMs mass, indicating that the polymer matrix constitutes the major component. Intriguingly, compared to the inherently high inorganic mass fractions of close-packed colloidal systems, this relatively low residual percentage, together with the high polymer content, serves as evidence for the non-close-packed spatial arrangement of the nanoparticles. Fundamentally, this structural feature endows the polymer network with abundant intrinsic free volume, accommodating unrestricted expansion and contraction, which may enable a broad range of optical tunability.

**Figure 3:**
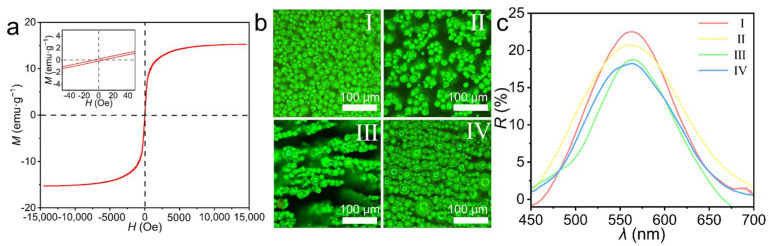
Magnetic properties and angle-independent structural color of MPCMs. (**a**) VSM hysteresis loop of MPCMs at room temperature. (**b**) Optical microscopy images of MPCMs under different magnetic field conditions: (**I**) without magnetic field; (**II**) perpendicular; (**III**) 45°; (**IV**) parallel. (**c**) Corresponding reflection spectra of MPCMs under the four conditions.

The vibrating sample magnetometer (VSM) hysteresis loop (Figure 3a) exhibits near-zero coercivity and remanence, indicating the typical superparamagnetic nature of the MPCMs. The saturation magnetization (*M*_s_) of the MPCMs is determined to be 15.2 emu/g. Compared to the pristine Fe_3_O_4_@TA nanoparticles (*M*_s_ = 40.3 emu/g, Figure S10), this expected reduction in magnetization is attributed to the substantial mass contribution of the non-magnetic hydrogel matrix and the relatively low volume fraction of the magnetic units, a direct consequence of their non-close-packed spatial arrangement. To evaluate their optical stability under external magnetic manipulation, the MPCMs were dispersed in ethanol and observed via optical microscopy (Figure 3b). Conventional one-dimensional magnetic photonic crystals and Janus-structured magnetic microspheres rely on continuous external magnetic fields to align the microspheres for a uniform color display, rendering them highly energy-consuming, inconvenient, and equipment-dependent. In contrast, the MPCMs inherently display a uniform and brilliant structural color even in the absence of any applied field (State I). Furthermore, when subjected to magnetic fields oriented perpendicular (State II), at a 45° angle (State III), or parallel (State IV) to the microscopic observation plane, the MPCMs exhibit rapid directional motion along the magnetic field lines. Remarkably, owing to their structural isotropy, their brilliant coloration remains visually unaltered throughout these dynamic spatial manipulations. This optical stability is in agreement with the quantitative reflection spectra (Figure 3c), where the characteristic reflection peak remains at 564 nm without any discernible shift, regardless of the applied field orientation. Ultimately, these results demonstrate that the MPCMs integrate magnetic responsiveness with angle-independent structural coloration, realizing the decoupling of magnetic actuation and optical display.

**Figure 4:**
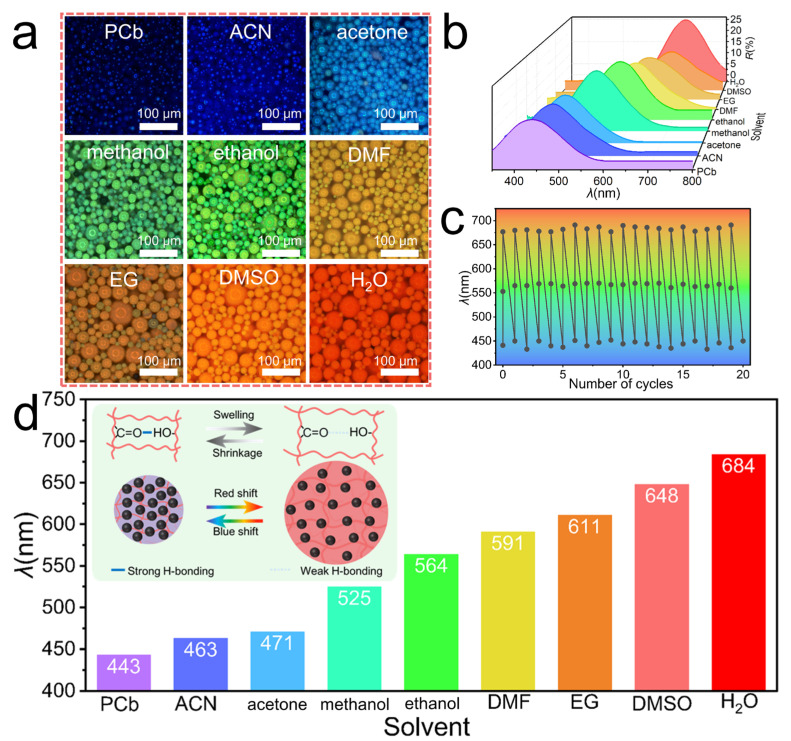
Solvent-responsive properties of MPCMs. (**a**) Optical microscopy images of MPCMs in nine typical solvents: PCb, ACN, acetone, methanol, ethanol, DMF, EG, DMSO, and H_2_O. (**b**) Corresponding reflection spectra. (**c**) Cyclic solvent exchange test of MPCMs among H_2_O, ethanol, and PCb over 20 cycles. (**d**) Reflection peak wavelengths of MPCMs in the nine solvents, with an inset showing the schematic illustration of the competitive hydrogen-bonding interactions governing the swelling and contraction of the polymer network.

Building on the non-close-packed architecture with intrinsic free volume and the demonstrated decoupling of optical response from magnetic actuation, the MPCMs provide an ideal platform to investigate purely solvent-driven photonic responses. As depicted in Figure 4a, immersing the MPCMs in nine typical solvents produces a continuous, full-spectrum color transition from purple (PCb) to red (H_2_O) [37]. The corresponding reflection spectra (Figure 4b) quantitatively confirm this continuous peak redshift, demonstrating spectral tunability for visual sensing. The sensitivity of the solvatochromic response is indicated by the continuous peak shift of 241 nm (from 443 nm to 684 nm) across the nine solvents. To evaluate reversibility, the MPCMs were subjected to cyclic solvent exchange among H_2_O, ethanol, and PCb, with negligible degradation in optical response after 20 cycles, demonstrating reversibility and durability for repeated operation. Macroscopic photographs of the MPCMs in PCb, ethanol, and H_2_O are provided in Figure S11. The structural coloration of the MPCMs originates from the non-close-packed assembly of the Fe_3_O_4_@TA nanoparticles, where the reflection peak wavelength (*λ*) is directly proportional to the average interparticle distance (*d*_avg_) according to Bragg’s law [32]. This distance is governed by the swelling state of the hydrogel network, which is dictated by competitive hydrogen-bonding interactions together with solvent viscosity, molecular size, and polymer–solvent affinity, where hydrogen bonding plays the dominant role. When the hydrogen-bonding interaction between the solvent and the polymer network is weak, interchain hydrogen bonding between the hydroxyl groups (-OH) of HEMA and the carbonyl groups (C=O) of NVP dominates, leading to network contraction, reduced *d*_avg_, and a blueshift of *λ*. Conversely, when the solvent forms strong hydrogen bonds with the polymer matrix, these interchain interactions are disrupted, inducing network swelling, increasing *d*_avg_, and resulting in a redshift. This swelling-shrinking transition is fully reversible, providing a reliable basis for solvent-dependent visual sensing (Figure 4d, inset).

To quantitatively elucidate these underlying interactions, the Kamlet–Taft solvatochromic parameter system was employed (Table S1), where *α* represents the solvent’s hydrogen-bond-donating ability and *β* denotes its hydrogen-bond-accepting ability [38]. Given that the copolymer network contains both hydrogen-bond donors (-OH from HEMA) and acceptors (C=O from NVP), solvents with high *α* preferentially interact with C=O groups, whereas those with high *β* tend to bind with -OH groups. Figure 4d summarizes the variation in *λ* across the nine solvents, and a clear correlation with their *α* and *β* values can be observed. PCb exhibits the lowest parameters (*α* = 0, *β* = 0.40), resulting in minimal solvent–polymer interaction, maximum network contraction, and the shortest *λ* (443 nm). Although ACN shares an identical *β* value to PCb, its slight hydrogen-donating ability (*α* = 0.19) enables weak interaction with the C=O groups, leading to a modest redshift to 463 nm. The slightly higher *β* of acetone (0.48) enhances its interaction with -OH groups, shifting *λ* to 471 nm. Notably, while both methanol and ethanol possess high *α* and *β* values, their resulting *λ* values (525 nm and 564 nm) are lower than expected. This deviation may be attributed to solvent self-association, which reduces the availability of hydrogen-bonding sites for interaction with the polymer network. Methanol, due to its smaller molecular size and higher hydroxyl density, exhibits stronger self-association than ethanol, further limiting effective interactions [39]. In contrast, DMF, an aprotic solvent with high *β* (0.69) but zero *α*, avoids such self-association effects and interacts efficiently with -OH groups, resulting in a larger redshift (591 nm). Furthermore, EG possesses high *α* (0.90) and *β* (0.52), which theoretically should induce pronounced swelling. However, its *λ* reaches only 611 nm, primarily due to its high viscosity (~16 mPa·s), which limits solvent diffusion and polymer chain relaxation. In comparison, DMSO exhibits the highest *β* value (0.76) among the tested organic solvents and relatively low viscosity, promoting stronger interactions with -OH groups and facilitating more extensive swelling, resulting in *λ* = 648 nm. Ultimately, H_2_O, with the highest *α* value (1.17) and moderate *β* (0.47), forms strong hydrogen bonds with both C=O and -OH groups, leading to maximal network swelling and the highest peak wavelength at 684 nm.

**Figure 5:**
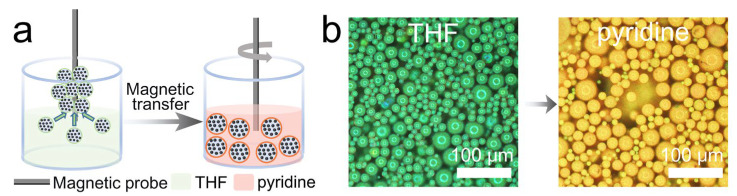
Magnetic transfer and solvent exchange of MPCMs for toxic solvent detection. (**a**) Schematic illustration of the magnetic transfer process. (**b**) Optical microscopy images of MPCMs before and after solvent exchange from THF to pyridine.

In the visual detection of toxic or hazardous solvents, conventional methods often involve substantial operational risks, while the detection media are typically difficult to recycle. The intrinsic magnetic responsiveness of the MPCMs provides a practical and effective strategy to address these limitations. Notably, both THF and pyridine are known to exhibit reproductive toxicity, making them representative hazardous solvents for validating this approach [40,41]. As illustrated in Figure 5a, a magnetic probe is used to collect MPCMs initially dispersed in THF. The MPCMs rapidly accumulate at the probe tip, forming a localized high-density region that enables rapid and contactless separation from the surrounding hazardous environment. Subsequently, by directly immersing the MPCMs-loaded probe into pyridine, combined with solvent wetting and mild mechanical agitation, the MPCMs readily detach and redisperse into the new solvent. This solvent exchange process induces a pronounced structural color transition from green to orange-yellow (Figure 5b). Reflection spectra recorded before and after solvent exchange for three independent magnetic transfer cycles (Figure S12) show a consistent peak shift from 552 nm to 581 nm. In the THF spectra, the reflectance at the peak position decreased from 22.7% in the first cycle to 17.9% in the third cycle, which may be attributed to slight loss of MPCMs during magnetic transfer. This shift originates from the difference in β between THF (0.55) and pyridine (0.64), leading to distinct swelling degrees, thereby altering the interparticle spacing and ultimately modulating the Bragg reflection wavelength. Benefiting from their excellent cyclic stability and solvent compatibility, the MPCMs enable reusable and minimally invasive detection across different solvent environments. This magnetically assisted manipulation significantly reduces exposure risks, thereby establishing a safer and more sustainable approach for the visual sensing of hazardous liquids.

**Figure 6:**
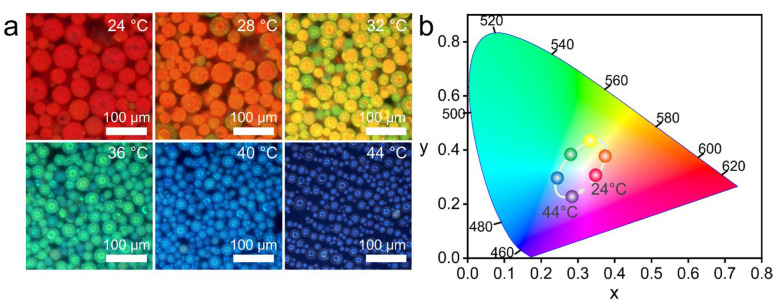
Thermo-responsive properties of PNIPAM-based MPCMs. (**a**) Optical microscopy images of MPCMs at temperatures ranging from 24 °C to 44 °C (24, 28, 32, 36, 40, 44 °C). (**b**) CIE chromaticity diagram showing the color evolution of MPCMs with increasing temperature.

In addition to the solvent-responsive MPCMs, a thermo-responsive version was also prepared by incorporating PNIPAM as a component into the MPCMs system. SEM reveals spherical morphology and a non-close-packed internal structure (Figure S13a,b). From TGA (Figure S13c), the Fe_3_O_4_ content in the thermo-responsive MPCMs is determined to be 50.9 wt%, referencing the pristine Fe_3_O_4_@TA nanoparticles (77.28% inorganic residue, Figure S9). VSM further confirms superparamagnetism with *M*_s_ of 20.9 emu/g and negligible remanence (Figure S13d). In this system, PCb served as a polar aprotic solvent with a high dielectric constant (~65) to promote electrostatic self-assembly of the nanoparticles, while also providing a homogeneous medium for NIPAM and other components. As shown in Figure 6a, increasing the temperature from 24 °C to 44 °C induces a continuous structural color transition from red to purple. The gradual color change is also clearly visible to the naked eye, as shown in the vial photographs in Figure S13e. This evolution is quantitatively represented on the CIE chromaticity diagram (Figure 6b), where the chromaticity coordinates trace a continuous trajectory across the color space. Correspondingly, the reflection peak exhibits a pronounced blueshift from 705 nm to 450 nm, spanning almost the entire visible spectrum (Figure S13f). Direct measurement of microsphere diameters from optical microscopy images at 24 °C and 44 °C showed that the average diameter decreased from 33.8 μm to 20.3 μm, corresponding to a linear shrinkage of approximately 40%. This value is close to the lattice contraction (≈36%) derived from Bragg’s law. Notably, this over 250 nm tuning range exceeds the typical spectral shifts (<170 nm) reported for conventional thermo-responsive photonic crystal microspheres [42,43,44], as summarized in Table S2. The dynamic temperature response was evaluated by rapidly transferring the MPCMs between 24 °C and 44 °C using a magnetic probe (Figure S13g). Upon heating, the reflection peak reached a new equilibrium within 26 s; upon cooling, it recovered within 44 s. Thermo-responsive behavior originates from two distinct factors. First, the PNIPAM network undergoes a volume phase transition near its lower critical solution temperature (~32 °C), providing the thermodynamic driving force for lattice contraction with changing temperature. Second, the non-close-packed architecture provides intrinsic free volume that allows lattice contraction without steric hindrance from neighboring particles, thereby enabling wide-range structural color modulation.

## 4. Conclusions

In summary, we developed MPCMs based on non-close-packed, optically isotropic colloidal assemblies. Monodisperse Fe_3_O_4_@TA nanoparticles self-assemble into non-close-packed amorphous structures driven by electrostatic repulsion and are subsequently fixed within stimuli-responsive polymer networks. Owing to their structural isotropy, the MPCMs exhibit angle-independent structural coloration, eliminating the need for continuous magnetic field alignment during optical readout and enabling passive visual sensing. The non-close-packed architecture provides sufficient free volume to accommodate isotropic expansion and contraction of the polymer matrix, resulting in a broad spectral tunability. Consequently, the MPCMs show optical responses to both solvent- and thermo-stimuli, with thermally induced wavelength shifts exceeding 250 nm. In addition, magnetic responsiveness allows for remote manipulation, enabling rapid collection, transfer, and reuse without perturbing the optical signal.

Despite these advantages, several aspects require further investigation. Long-term structural and chemical stability under operational and storage conditions remains to be established. In addition, the performance of these systems in complex environments, where ionic strength, multicomponent interactions, and rheological conditions may affect their responsiveness, should be systematically evaluated. Finally, improving particle uniformity and developing scalable fabrication strategies will be critical for enabling MPCMs toward real-world applications. Overall, this work provides a strategy for constructing isotropic, stimuli-responsive MPCMs, opening new opportunities for advanced optical sensing, smart displays, and adaptive photonic devices.

## Data Availability

The original contributions presented in the study are included in the article/Supplementary Materials; further inquiries can be directed to the corresponding authors.

## Supplementary Materials

The following supporting information can be downloaded at https://www.mdpi.com/article/10.3390/nano16090556/s1, Figure S1: ζ-potential of Fe_3_O_4_@TA nanoparticles dispersed in ethanol. Section S1: Preparation of Fe_3_O_4_@TA core–shell nanoparticles. Figure S2: Chemical structures of the polymers used in this work. (**a**) P(HEMA-*co*-NVP); (**b**) PNIPAM. Figure S3: Optical microscopy image of MPCMs prepared without NVP (using only HEMA) after dispersion in ethanol. Figure S4: (**a**) Optical microscopy image of the droplets; (**b**) size distribution of droplets. Figure S5: Optical microscopy images of MPCMs dispersed in ethanol after curing at 80 °C for (**a**) 3 min, (b) 5 min, and (**c**) 7 min. Figure S6: (**a**) SEM image and (**b**) size distribution of Fe_3_O_4_@TA nanoparticles. Figure S7: (**a**) Optical microscopy image of MPCMs dispersed in ethanol and (**b**) size distribution of MPCMs. Figure S8: Center-to-center distance distribution between adjacent Fe_3_O_4_@TA nanoparticles measured from high-magnification SEM images. Figure S9: TGA curves of Fe_3_O_4_@TA nanoparticles. Figure S10: VSM hysteresis loop of Fe_3_O_4_@TA nanoparticles measured at room temperature. Figure S11: Vial photographs of solvent-responsive MPCMs in PCb, ethanol, and H_2_O. Figure S12: Reflection spectra of MPCMs in THF and pyridine recorded before and after solvent exchange for three independent magnetic transfer cycles. Figure S13: (**a**) SEM images of thermos-responsive MPCMs. (**b**) Cross-sectional SEM images at higher magnification. (**c**) TGA curve. (**d**) VSM hysteresis loop. (**e**) Vial photographs of MPCMs at 24 °C and 44 °C. (**f**) Reflection spectra of MPCMs at 24 °C and 44 °C. (**g**) Reflection peak wavelength during heating and cooling. Table S1: Kamlet–Taft parameters (α and β) of the solvents used in this study, where α represents the hydrogen-bond donating ability and β represents the hydrogen-bond accepting ability. Table S2: Comparison of thermo-responsive photonic crystal microspheres.
