# Supplementary material for: Non-Close-Packed Isotropic Responsive Magnetic Photonic Crystal Microspheres
Source: Nanomaterials (Basel). 2026 May 1;16(9):556. doi: 10.3390/nano16090556 (PMC13164807; doi:10.3390/nano16090556)
Supplement: Supplementary file 1 [file nanomaterials-16-00556-s001.zip › nanomaterials-4257521-supplementary.pdf]

Non-close-packed Isotropic Responsive Magnetic Photonic Crystal Microspheres

Lejian Zhao [1], Jie Zhu [2], Maocheng Sun [2], Wei Luo [2,*], Huiru Ma [3,*] and Jianguo Guan [1,4]

1 State Key Laboratory of Advanced Technology for Materials Synthesis and Processing, International School of Materials Science and Engineering, Wuhan University of Technology, Wuhan 430070, China; 298552@whut.edu.cn (L.Z.); guanjg@whut.edu.cn (J.G.)
2 School of Materials Science and Engineering, Wuhan University of Technology, Wuhan 430070, China; 345097@whut.edu.cn (J.Z.); 1209061600@qq.com (M.S.)
3 School of Chemistry, Chemical Engineering and Life Science, Wuhan University of Technology, Wuhan 430070, China
4 Wuhan Institute of Photochemistry and Technology, 7 North Bingang Road, Wuhan 430083, China
* Correspondence: rowell@whut.edu.cn (W.L.); mahr@whut.edu.cn (H.M.)

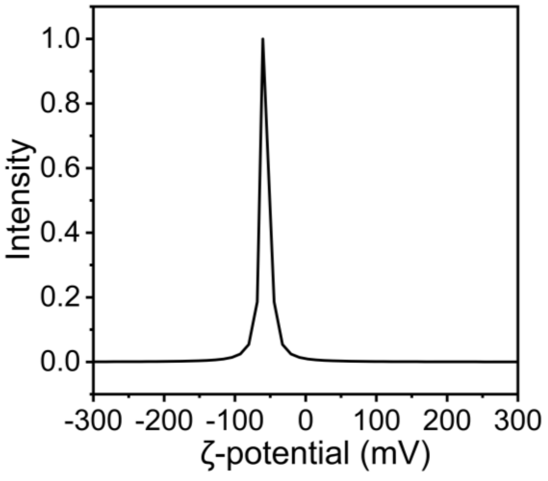

Figure S1. ζ-potential of Fe_3O_4@TA nanoparticles dispersed in ethanol.

Section S1. Preparation of Fe_3O_4@TA Core–Shell Nanoparticles.

Fe_3O_4 nanoparticles were synthesized according to our previous work[35]. After synthesis, the nanoparticles were washed five times with ethanol using magnetic separation to remove impurities and then dispersed in ethanol to obtain a stock dispersion of 100 mg/mL. A 200 μL aliquot of this dispersion was mixed with 10 mg of tannic acid (TA), ultrasonicated for 10 min until homogeneous, and then allowed to stand for 30 min to ensure sufficient adsorption of TA. The product was collected by centrifugation, washed five times with deionized water and five times with ethanol to remove residual TA, and finally re-dispersed in 1 mL of ethanol to yield a Fe_3O_4@TA dispersion with a concentration of 20 mg/mL. Detailed parameters for the synthesis of Fe_3O_4 can be found in [35], and for the TA coating in [11].

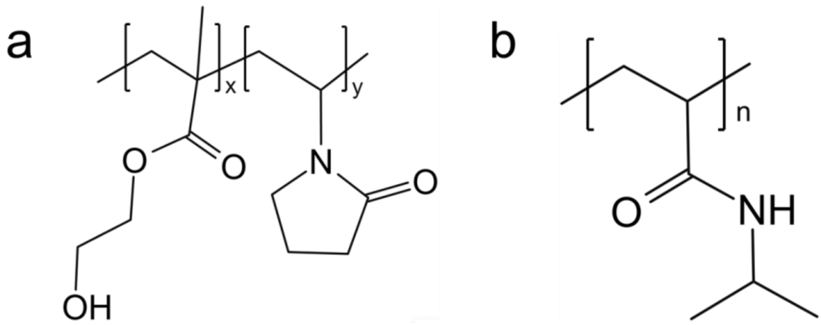

Figure S2. Chemical structures of the polymers used in this work. (a) P(HEMA-*co*-NVP). (b) PNIPAM.

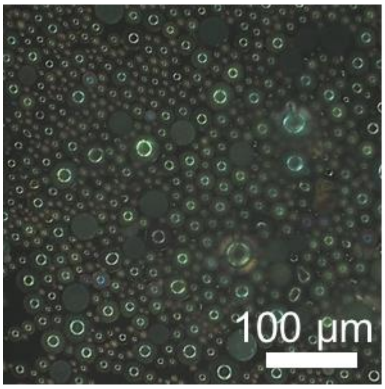

Figure S3. Optical microscopy image of MPCMs prepared without NVP (using only HEMA) after dispersion in ethanol.

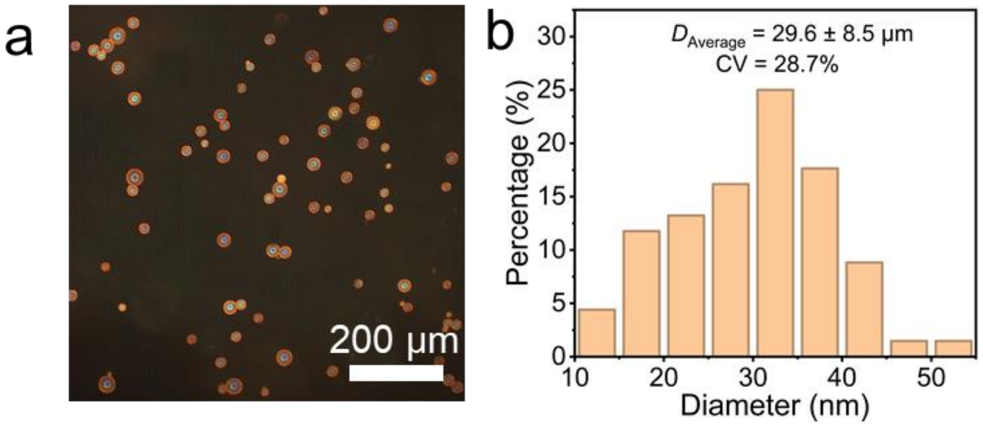

Figure S4. (a) Optical microscopy image of the droplets. (b) Size distribution of droplets.

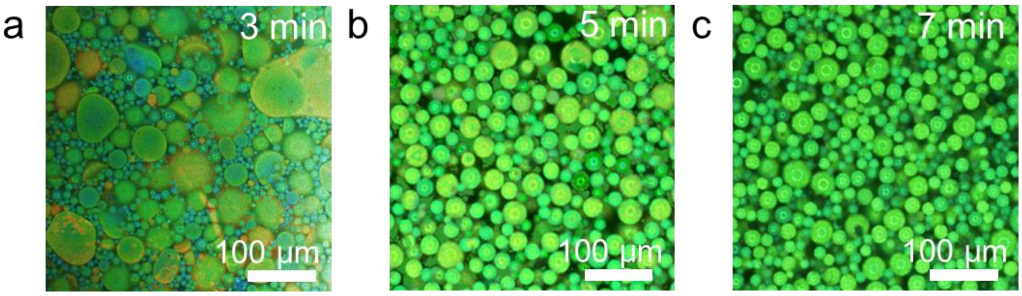

Figure S5. Optical microscopy images of MPCMs dispersed in ethanol after curing at 80 °C for (a) 3 min, (b) 5 min, and (c) 7 min.

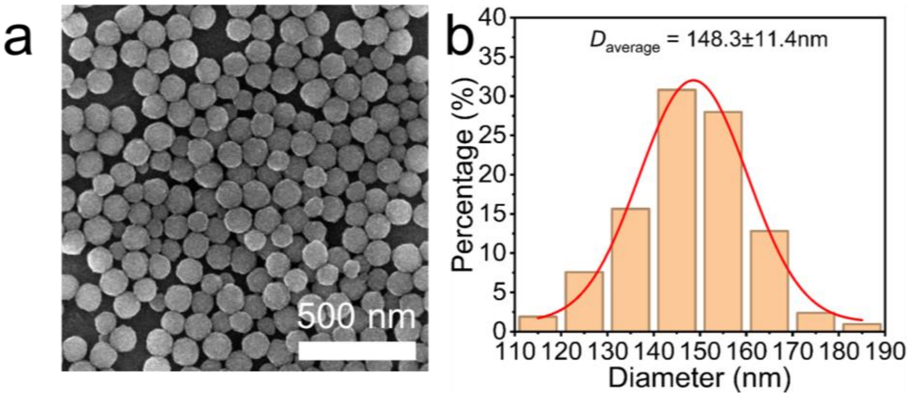

Figure S6. (a) SEM image. (b) Size distribution of Fe_3O_4@TA nanoparticles.

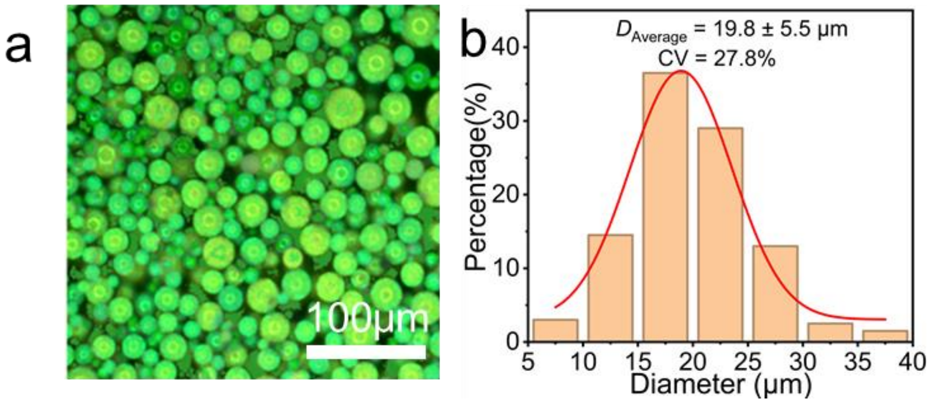

Figure S7. (a) Optical microscopy image of MPCMs dispersed in ethanol. (b) Size distribution of MPCMs.

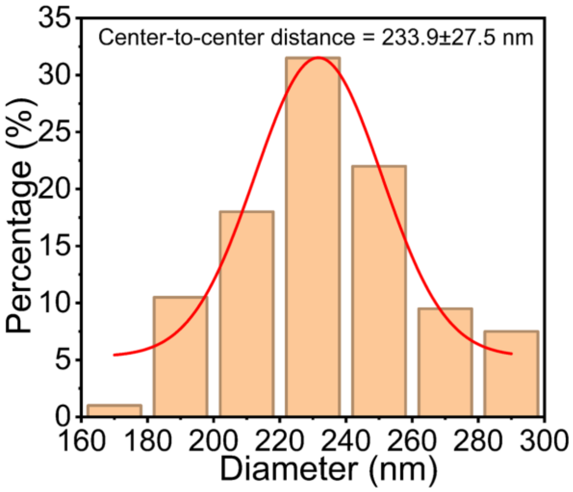

Figure S8. Center-to-center distance distribution between adjacent Fe_3O_4@TA nanoparticles measured from high-magnification SEM image.

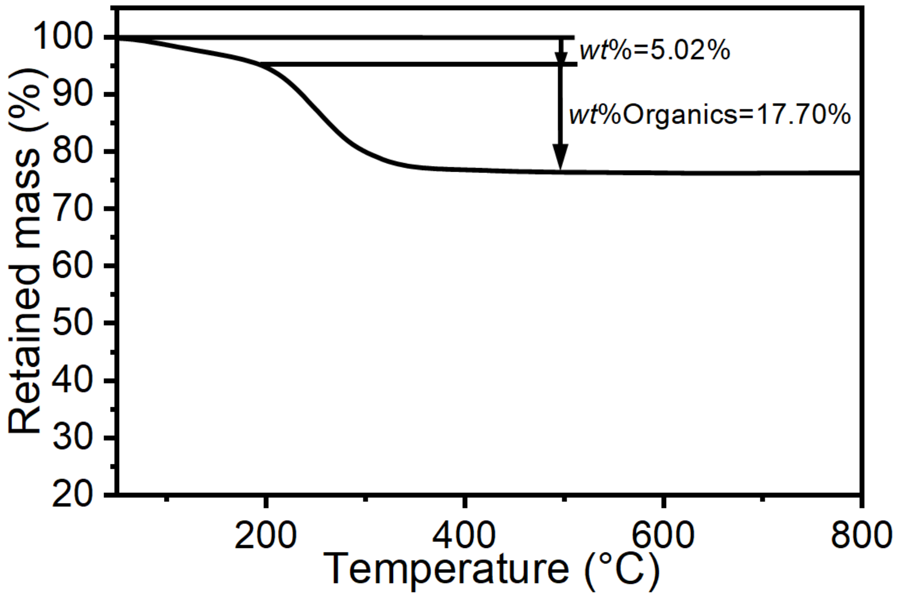

Figure S9. TGA curves of Fe_3O_4@TA nanoparticles.

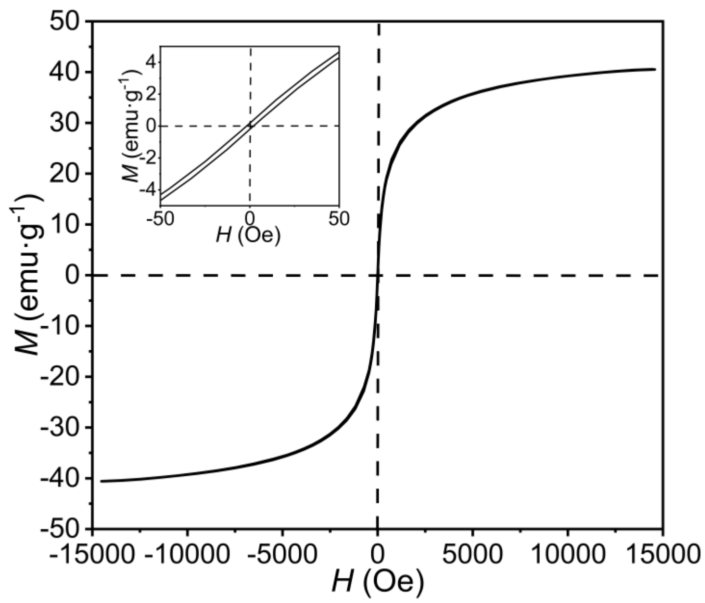

Figure S10. VSM hysteresis loop of Fe_3O_4@TA nanoparticles measured at room temperature.

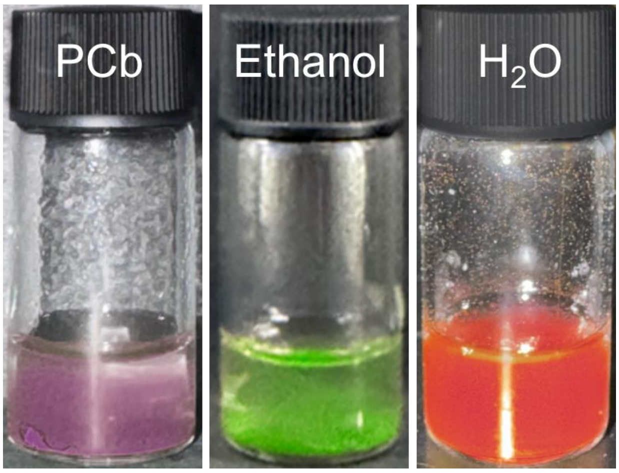

Figure S11. Vial photographs of solvent-responsive MPCMs in PCb, ethanol, and H_2O.

Table S1. Kamlet–Taft parameters (α and β) of the solvents used in this study, where α represents the hydrogen-bond donating ability and β represents the hydrogen-bond accepting ability.

Solvent	α	β
PCb	0	0.40
ACN	0.19	0.40
acetone	0.08	0.48
methanol	0.93	0.62
THF	0	0.55
ethanol	0.83	0.77
pyridine	0	0.64
DMF	0	0.69
EG	0.90	0.52
DMSO	0	0.76
H_2O	1.17	0.47

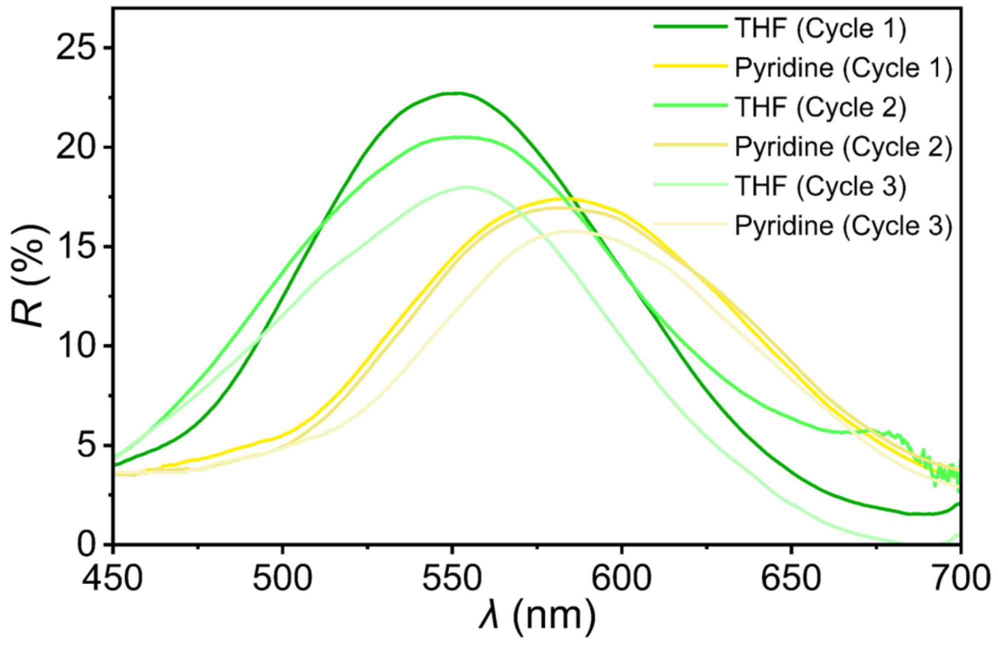

Figure S12. Reflection spectra of MPCMs in THF and pyridine recorded before and after solvent exchange for three independent magnetic transfer cycles.

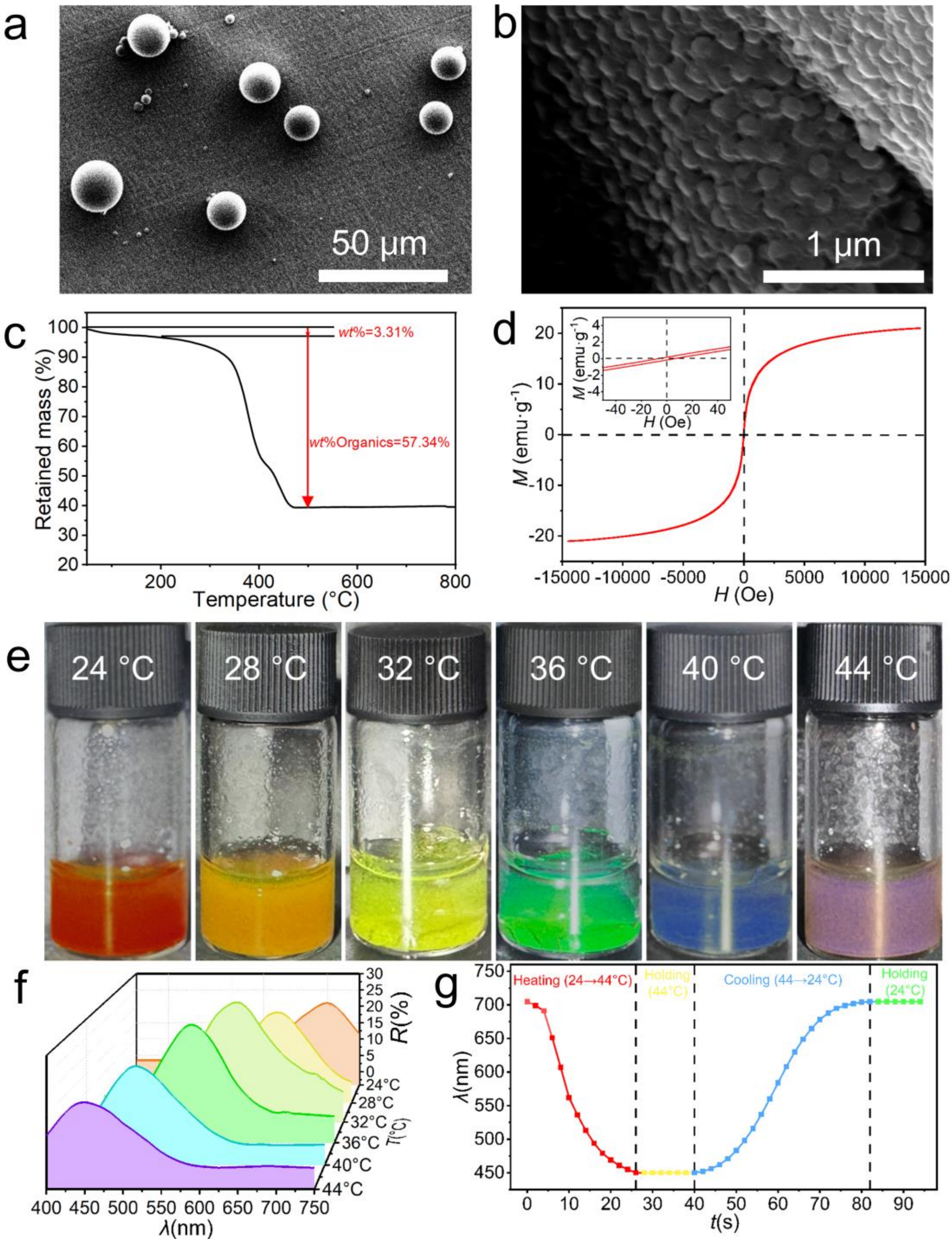

Figure S13. (a) SEM images of thermo-responsive MPCMs. (b) Cross-sectional SEM images at higher magnification. (c) TGA curve. (d) VSM hysteresis loop. (e) Vial photographs of MPCMs at 24 °C and 44 °C. (f) Reflection spectra of MPCMs at 24 °C and 44 °C. (g) Reflection peak wavelength during heating and cooling.

Table S2. Comparison of thermo-responsive photonic crystal microspheres.

Reference	Polymer	Microsphere size (μm)	Temperature range (°C)	Tuning range (nm)
Wang et al.[42]	P(NIPAM-co-MAA)	~100 (est.)	20–45	~180
Luo et al.[43]	PNIPAM	~400	10–35	~90
Jia et al.[44]	P(NIPAM-co-AM)	~620	10–50	~170
This work	PNIPAM	~20	24–44	>250
